# Aging does not Enhance Experimental Cigarette Smoke-Induced COPD in the Mouse

**DOI:** 10.1371/journal.pone.0071410

**Published:** 2013-08-02

**Authors:** Steven Zhou, Joanne L. Wright, Joseph Liu, Don D. Sin, Andrew Churg

**Affiliations:** 1 Department of Pathology, University of British Columbia, Vancouver, British Columbia, Canada; 2 Department of Medicine (Respiratory Division) and the UBC James Hogg Research Center, University of British Columbia, Vancouver, British Columbia, Canada; National Heart and Lung institute, United Kingdom

## Abstract

It has been proposed that the development of COPD is driven by premature aging/premature senescence of lung parenchyma cells. There are data suggesting that old mice develop a greater inflammatory and lower anti-oxidant response after cigarette smoke compared to young mice, but whether these differences actually translate into greater levels of disease is unknown. We exposed C57Bl/6 female mice to daily cigarette smoke for 6 months starting at age 3 months (Ayoung@) or age 12 months (Aold@), with air-exposed controls. There were no differences in measures of airspace size between the two control groups and cigarette smoke induced exactly the same amount of emphysema in young and old. The severity of smoke-induced small airway remodeling using various measures was identical in both groups. Smoke increased numbers of tissue macrophages and neutrophils and levels of 8-hydroxyguanosine, a marker of oxidant damage, but there were no differences between young and old. Gene expression studies using laser capture microdissected airways and parenchyma overall showed a trend to lower levels in older animals and a somewhat lesser response to cigarette smoke in both airways and parenchyma but the differences were usually not marked. Telomere length was greatest in young control mice and was decreased by both smoking and age. The senescence marker p21^Waf1^ was equally upregulated by smoke in young and old, but p16^INK4a^, another senescence marker, was not upregulated at all. We conclude, in this model, animal age does not affect the development of emphysema and small airway remodeling.

## Introduction

Chronic obstructive lung disease (COPD) consists of 4 anatomic lesions: emphysema, small airway remodeling (SAR), vascular remodeling associated with pulmonary hypertension, and mucus metaplasia/hypertrophy of bronchial mucus glands associated with mucus hypersecretion (clinical chronic bronchitis).

The pathogenesis of all of these lesions is poorly understood and is the subject of extensive investigation. A number of different theories of pathogenesis have been developed, and one of these is the idea that COPD, or at least emphysema, represents premature aging/premature senescence of lung parenchymal cells, induced in part by oxidative damage from cigarette smoke components [1]–[6], and that small airway remodeling reflects senescence of Clara cells [7]. A variety of markers of aging/senescence have been shown to be upregulated in human and experimental COPD ([1]–[6], and see Discussion).

One of the implications of this hypothesis is that older individuals should show greater susceptibility to the effects of cigarette smoke, but this issue has received little direct examination. It has been reported that older mice develop a greater inflammatory response, either spontaneously or after smoke exposure, compared to young mice ([8]–[10], and see Discussion), and conversely, a decreased antioxidant response [10], [11]. However, whether older animals actually develop worse COPD compared to younger has not been examined.

In this study we exposed relatively young (3 months old at start of exposure) and relatively old (12 months old at start of exposure) mice to cigarette smoke for 6 months in an attempt to see if older mice developed more severe COPD and had differences in inflammation and expression of structural genes, inflammatory mediators and antioxidant/detoxification genes, and levels of oxidant damage, as well as putative markers of aging/senescence.

## Materials and Methods

### Animals and Smoke Exposures

The experiments described here were approved by the Animal Care Committee of the University of British Columbia. Groups of 5 to 7 C57Bl/6 female mice (Charles River, Montreal, Canada) were exposed to cigarette smoke or air (control) starting at age 3 months (Ayoung mice@). An additional set of 6 month old mice (the oldest available) was purchased from Charles River and maintained in our animal facility until age 12 months (Aold mice@); they were then exposed to cigarette smoke or air for 6 months. Animal purchase was timed so that smoke exposures for young and old mice were concomitant and consisted of the whole smoke from a mixture of three 2R1/2R4F Kentucky Research cigarettes 5 days per week for 6 months using a nose only exposure apparatus [12]. The 6 month smoke exposure was chosen because this is the minimal amount of smoke exposure that produces reliable signals for emphysema and small airway remodeling in C57Bl/6 mice [13]. Female mice were used because, in our experience, they get more severe disease than male mice. Control animals were handled, but not exposed to smoke. The animals were housed on standard bedding and allowed free access to chow and water. “Young” animals were 9 months old at the time of sacrifice and “old” animals 18 months old.

### Morphometric Analyses

Animals were sacrificed by CO_2_ overdose, and the lungs inflated with 0.6% agarose to 25 cm H_2_O pressure, using the methodology of Halbower et al [14]. Lungs were then fixed in formalin for 48 hours and sliced in a sagittal plane. Tissue was embedded in paraffin and cut at 5 microns thickness.

### Airspace Size

Histologic sections were cut at 5 microns thickness and stained with Hematoxylin and Eosin. Fifteen random fields were photographed. Using Image Pro ^(^™^)^ (Media Cybernetics, Silver Spring, MD), we measured mean linear intercept (Lm) and surface to volume ratio (S/V) using the following equations: Lm = 2 (length of line)/number of intercepts. S/V = 2 (ratio of test points to line length) X (intercepts/points).

### Small Airway Morphometry

Slides were stained with Picrosirius red and all rounded noncartilagenous airways were photographed. Using Image Pro, we measured internal (basement membrane to basement membrane) and external (adventitial border to adventitial border) bronchiolar diameter, and calculated wall thickness by subtracting the two measurements. Wall area was calculated as the area between the basement membrane and adventitial boundary. Collagen was calculated using color segmentation, selecting red values between 106 and 162. Wall area and collagen data were normalized to the perimeter of the epithelial basement membrane. The mean value for all airways in each animal was determined and used for statistical analysis.

### Immunohistochemical Staining for Neutrophils and Macrophages

Immunohistochemistry was carried out on the formalin fixed paraffin embedded tissue using Santa Cruz rat anti-mouse macrophage antibody sc-101447 and rat anti-mouse neutrophil antibody sc-71674 (Santa Cruz Biotechnology, Dallas TX), each diluted 1∶250 and incubated overnight at room temperature after antigen retrieval with pH 6.0 10 mM sodium citrate and heat. The second antibody was biotinylated anti-rat IgG 1∶200 (Vector Laboratory Inc. Burlingame CA BA-9401). Reaction product was visualized using streptavidin/HRP (Dako catalog P0397) and Nova Red (Vector Laboratory Inc). Numbers of macrophages and neutrophils were quantitated by counting 5 random high power fields and expressed as numbers of cells/mm^2^ of tissue.

### Immunohistochemical Staining for 8-hydroxyguanosine

Immunohistochemistry was carried out on the formalin fixed paraffin embedded tissue using Abcam (Toronto, Ontario) mouse monoclonal anti-8 hydroxyguanosine (Cat 62623). Sections were rehydrated, treated with Peroxidase 1 (BioCare, Concord, CA) for 3 minutes, and antigen retrieval performed with Rodent Decloaker (BioCare) using a steamer for 30 minutes, followed by a cooling step for 30 minutes. This was followed by a 30 minute incubation in Rodent Block M (Biocare), then a 2 hour incubation in primary antibody using primary antibody at 1∶250 diluted with a 50∶50 mixture of antibody diluent and Background Sniper (Biocare). Amplification and visualization were performed with a mouse on mouse polymer kit (BioCare) for 20 minutes and finally incubation with Vulcan Fast Red for 10 minutes.

For analysis of 8-deoxyguanosine staining of small airway (bronchiolar) nuclei, 5 airways from each animal were counted as follows. A point was placed in the center of the airway lumen and lines cast outward around the clock. A random number between 1 and 6 was generated by throwing dice. The clock line identified by the dice was identified and 5 nuclei on either side of the line point and its opposite clock were counted as either positive or negative for staining. The procedure was performed twice for each airway. Results were expressed as proportion of positive nuclei.

### Laser Capture Microdissection and RT-PCR Analysis of Bronchioles and Parenchyma

In an additional set of smoke or air-exposed mice, the lungs were inflated at sacrifice with OCT™ frozen section medium diluted 50% with normal saline, and immediately frozen in tissue cassettes at -80^o^ C. Frozen sections were cut at 5 μ thickness on membrane PEN slides (Leica, Concord, Ontario), and small airways (bronchioles) and parenchymal tissue collected by laser capture microdissection using a Leica LMD 6500 laser capture system. RNA was extracted using Arcturus PicoPure RNA Isolation kit (Applied Biosystems/Invitrogen, Foster City, CA) as per the manufacturer’s instructions. Reverse transcription of mRNA was carried out by using a High Capacity RNA-to-cDNA kit (Applied Biosystems/Invitrogen). Gene expression was determined by real-time PCR using TaqMan probes (Applied Biosystems) and an Applied Biosystems StepOnePlus machine. All samples for a given gene were run for young and old animals and airway and parenchymal samples at the same time. The amount of PCR product derived from each mRNA was normalized to that from β2-microglobulin in the same sample.

### Analysis of Telomere Length

DNA samples were extracted from blocks of formalin fixed and paraffin embedded lung tissue (5 animals per treatment group). The tissues were first de-waxed with xylene, and followed by two washes with 100% ethanol. The samples were then washed with 90% ethanol, followed by another wash with 70% ethanol, and then a final wash with 50% ethanol to rehydrate the tissue. After de-waxing, DNAeasy Blood and Tissue Kit (QIAGEN, Hilden, Germany) were used to extract DNA from the samples. Briefly, tissues were digested overnight at 56°C with 180 µl of ATL buffer and 20 µl proteinase K. After digestion, 200 µl of AL buffer was added and incubated at 70°C for 10 minutes, followed by ethanol precipitation. The solution was transferred into a spin column, and washed with the wash buffers provided in the kit. DNA was eluted with 200 µl of AE buffer. The concentration and purity of DNA were assessed using NanoDrop 2000 (NanoDrop Products, Wilmington, USA). All samples were diluted to a DNA concentration of 1ng/ul prior to use.

Telomere lengths of mouse lung tissue were measured using a qPCR protocol as described previously by Callicott and Womack [15]. In brief, the basis of this assay is to measure the amount of telomeric DNA per sample as a ratio to the total amount of genomic DNA, which is given by the measurement of acidic ribosomal phosphorprotein PO (36B4) gene. Telomeric forward and reverse primer sequences (Sigma, The Woodlands, TX) were (5′→3′) CGG TTT GTT TGG GTT TGG GTT TGG GTT TGG GTT TGG GTT and (5′→3′) GGC TGG CCT TAC CCT TAC CCT TAC CCT TAC CCT TAC CCT, respectively, while murine 36B4 forward and reverse primer sequences were (5′→3′) ACT GGT CTA GGA CCC GAG AAG and (5′→3′) TCA ATG GTG CCT CTG GAG ATT, respectively. Measurements were performed in triplicate per sample on 384-well Clear Optical Reaction Plates (Applied Biosystems, Foster City, CA). To serve as reference, a DNA sample extracted from the lung tissue of a C57BL/6J mouse was added to each plate. Five ng of both sample and reference DNA were added to wells and dried over-night within a biosafety cabinet. The reaction mixture per well included 10 µL QuantiTect SYBR Green PCR Master Mix (QIAGEN, Mississauga, ON), 8 uL UltraPure DNase/RNase-Free Water (Invitrogen), 1 uL of forward primer, and 1 uL of reverse primer, which yielded a final primer concentration of: tel fwd, 270 nM; tel rev, 900 nM; 36B4 fwd, 300 nM; 36B4 rev, 500 nM, and a final reaction volume of 20 uL. After loading, plates were sealed with MicroAmp Optical Adhesive Film (Applied Biosystems, Foster City, CA) and centrifuged briefly at 2,500 rpm for 2 minutes. The reactions were performed in an ABI ViiA 7 Real-time PCR System (Applied Biosystems, Foster City CA). The thermal cycling profile for the telomere portion of the assay was set at 95°C for 10 min followed by 30 cycles of 95°C for 15 s and 56°C anneal-extend step for 1 minute, with data collection. Cycling conditions for 36B4 were 95°C for 10 min, followed by 35 cycles of data collection at 95°C for 15 s, 52°C annealing step for 20 s, and extension step at 72°C for 30 s. Calculations for telomere length over single copy gene ratio (T/S ratio) were done according a method described by Cawthon [16]. The T/S ratio for each sample were compared to the T/S ratio of the reference DNA and expressed as relative telomere length.

### Statistics

Data were analyzed by analysis of variance with Tukey corrections for multiple comparisons except for relative telomere length comparisons between groups which were tested using a Wilcoxon Test, followed by Dunn multiple comparison post-test.

## Results

### Measures of Emphysema

**Figure 1:**
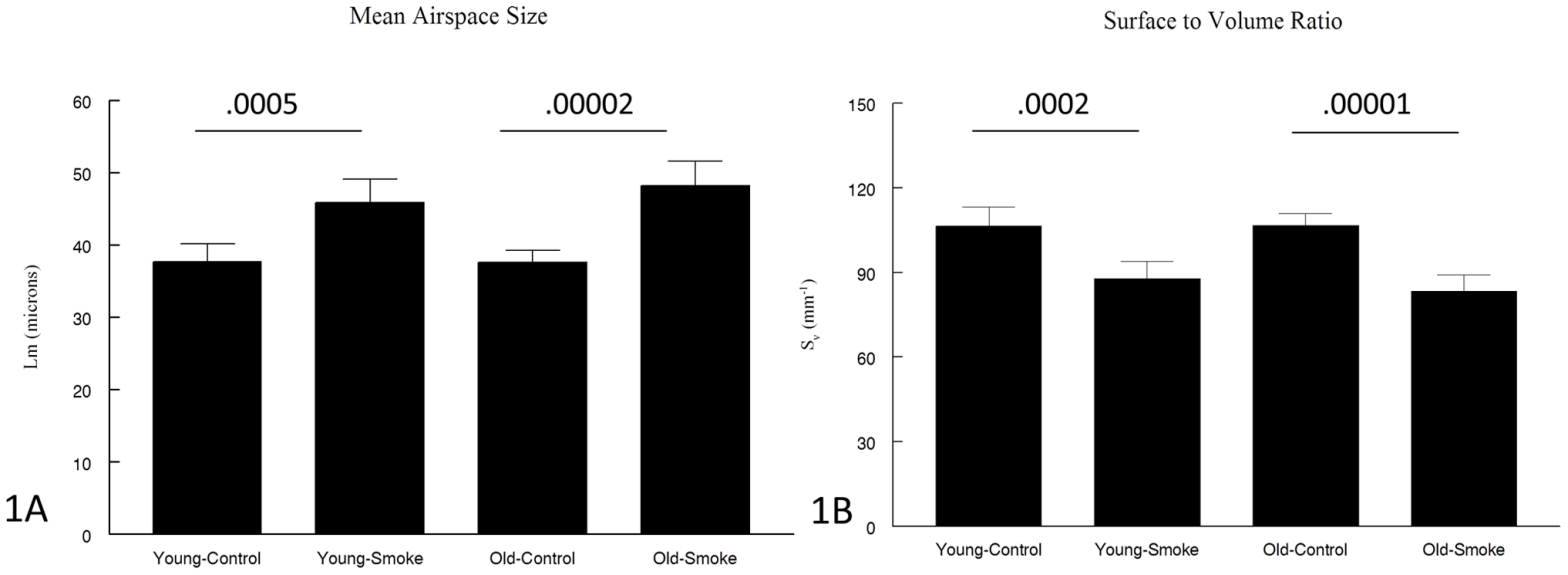
Measures of emphysema. 1A: Mean airspace size is increased by a similar amount in young and old animals. 1B. Surface to volume ratio is decreased by a similar amount in young and old animals. Data are mean ± SD. N = 5 to 7 animals/group. P values as shown.

Figure 1A shows data for mean airspace size (Lm) and Figure 1B data for surface to volume ratio (Sv). For both measures, the values in the control young and old mice exposed to air were identical. Smoke significantly increased Lm by 21.4% in the young mice and 28.1% in the old mice but the increase in the 2 groups was not statistically different. Similarly Sv was decreased by 18% in the young mice and 22% in the old mice, and the decreases were not statistically different between young and old.

### Measures of Small Airway Remodeling

**Figure 2:**
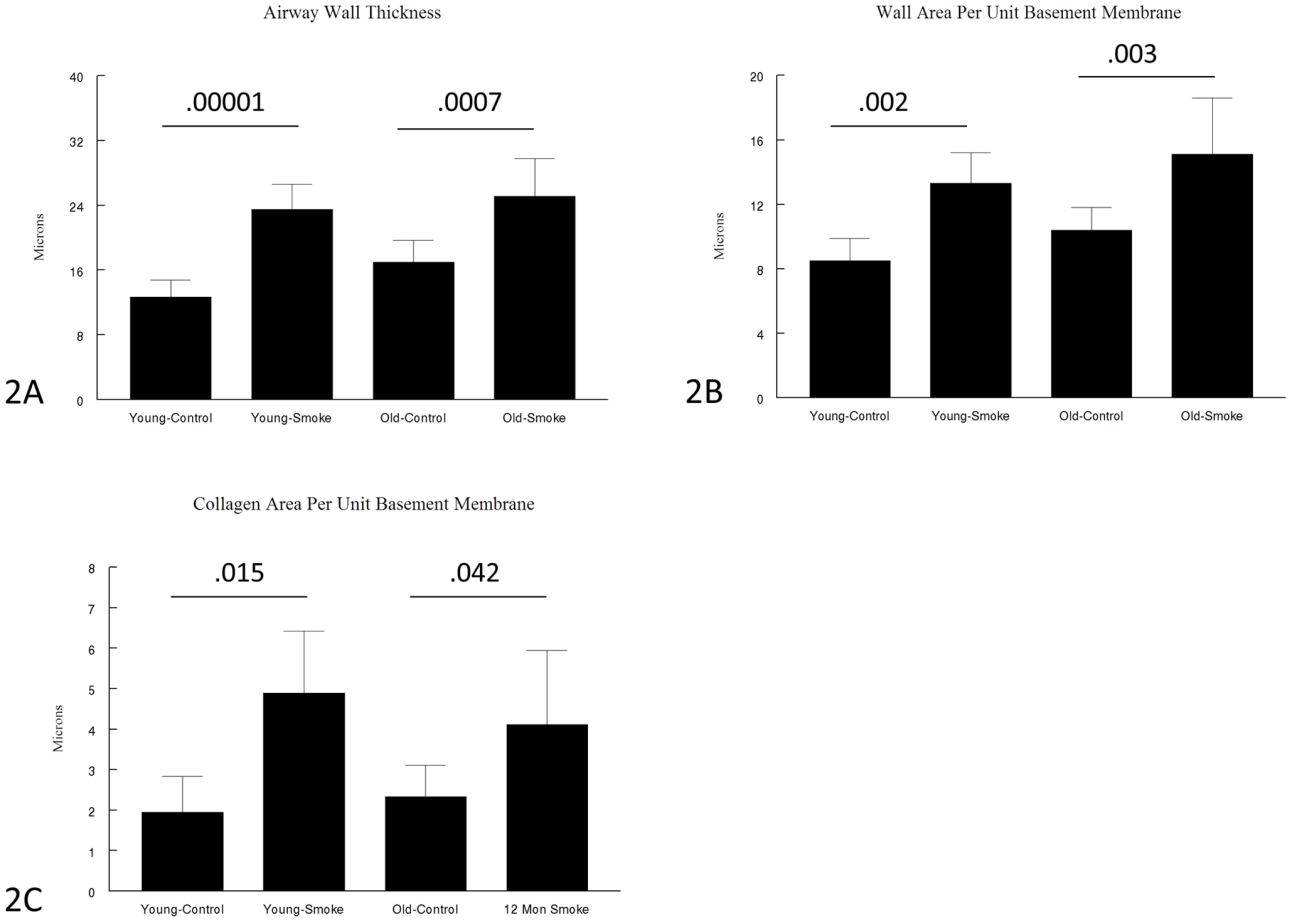
Measures of small airway remodeling. There are no differences in airway wall thickness (2A), wall area per unit basement membrane length (2B) or collagen area per unit basement membrane length (2C) comparing young and old animals. Data are mean ± SD. N = 5 to 7 animals/group. P values as shown.

Figure 2 shows data for wall thickness (2A), wall area/unit basement membrane length (2B), and collagen/unit basement membrane length (2C). For all 3 measures there was an increase in old mice exposed to air compared to young mice compared to air (34% for wall thickness, 22% for wall area, and 21% for collagen), but these values were not statistically different.

Smoke exposure caused a significant increase in all 3 measures in both young and old mice compared to control. Of these collagen was the most marked (2.6 times in young mice, 1.8 times in old mice), and the magnitude of the increase for all 3 measures was such that the old mice ended up with mean values for wall thickness, wall area, and collagen that were not statistically different from the values seen in the young mice.

### Inflammatory Cell Infiltration

**Figure 3:**
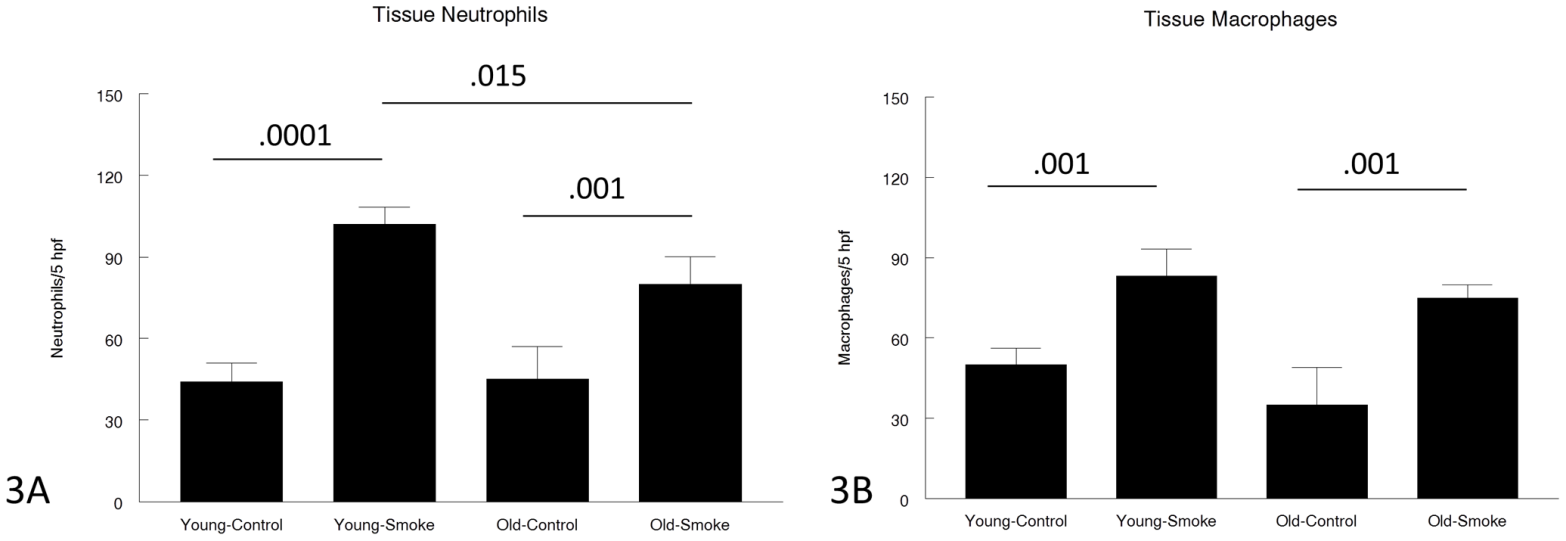
Tissue (parenchymal) inflammatory cells. Tissue neutrophils (3A) are increased by smoke in both young and old animals, but to a slightly lesser extent in old animals. Tissue macrophages (3B) are increased to the same extend by smoke in young and old animals. Data are mean ± SD. N = 5 animals/group. P values as shown.

Figure 3 shows neutrophil (3A) and macrophage (3B) cell counts/5 high power microscope fields. There were no differences between control young and old mice for either measure. Smoke exposure increased macrophage numbers in both age groups and there was no difference between 3 and 12 month animals. Smoke also increased tissue neutrophil numbers and the increase was slightly but significantly less in the 12 month compared to the 3 month animals.

### Airway Oxidant Damage Assessed by 8-hydroxyguanosine Staining

**Figure 4:**
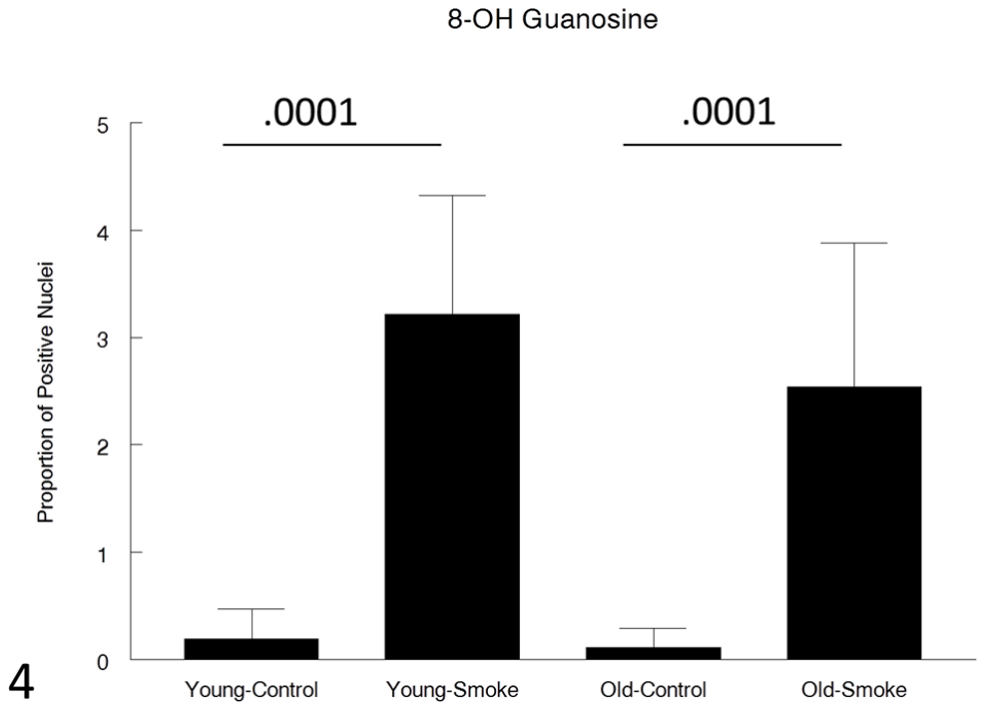
Small airway oxidative damage measured by 8-OH Guanosine staining. Smoke causes a similar increase in both young and old animals. Data are mean ± SD. N = 5 animals/group. P values as shown.

Levels of 8-hydroxyguanosine staining in bronchioles were very low in control animals and were not different between young and old (Figure 4). The proportion of staining nuclei was markedly increased (approximately 20 to 30 fold) in smokers, but again there were no differences between young and old (Figure 4).

### Gene Expression in Airways and Parenchyma

Using laser capture microdissection, we examined expression of genes relating to four broad areas: matrix production/breakdown; inflammatory response; anti-oxidant defense; and senescence, in airways and in parenchyma. Overall there was a trend towards reduced levels of expression with smoke exposure in old compared to young mice, and there were no instances in which gene expression was higher in old mice.

**Figure 5:**
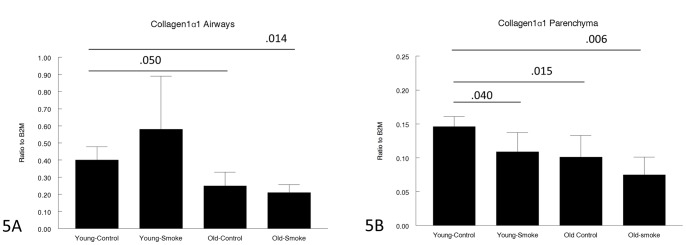
Gene expression of collagen 1α1 in laser capture microdissected airways (bronchioles) (5A) and parenchyma (5B). Levels of collagen 1α1 are lower in the old animals. Data are mean ± SD. N = 5 animals/group. P values as shown.

**Figure 6:**
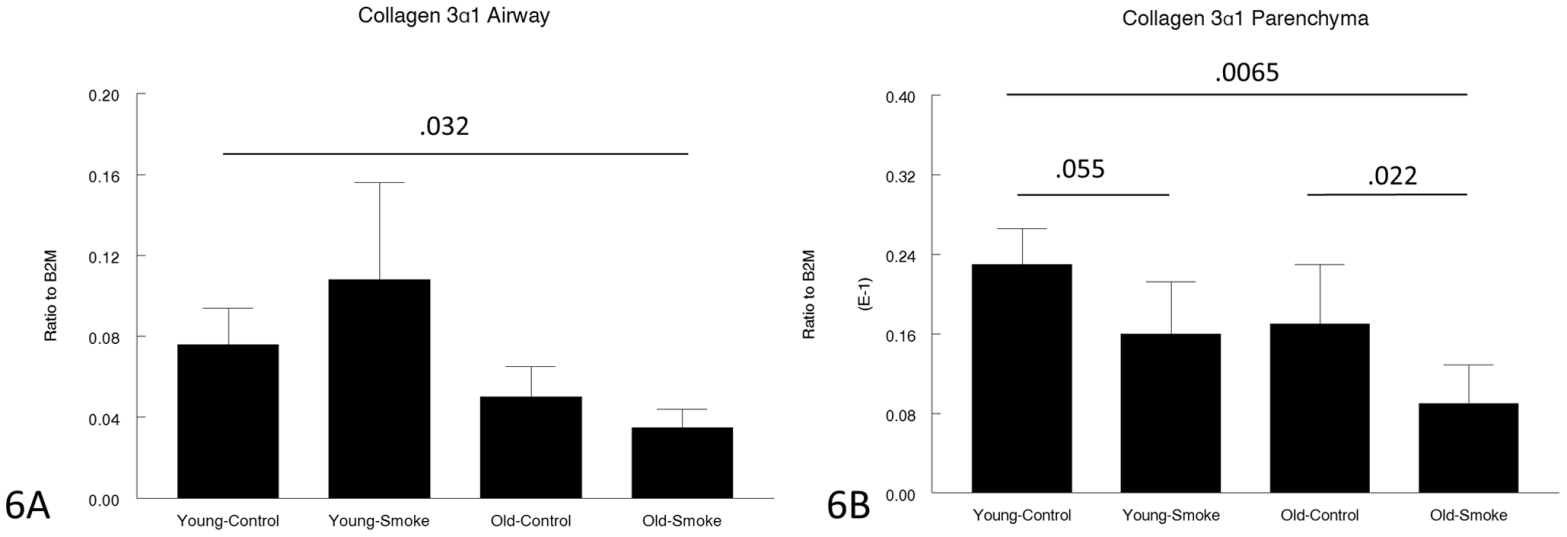
Gene expression of collagen 3α1 in laser capture microdissected airways (bronchioles) (6A) and parenchyma (6B). Levels of collagen 3α1 are lower in the old animals. The decrease in parenchymal collagen 3α1 with smoke exposure suggests that the parenchyma fails to repair smoke-induced damage. Data are mean ± SD. N = 5 animals/group. P values as shown.

**Figure 7:**
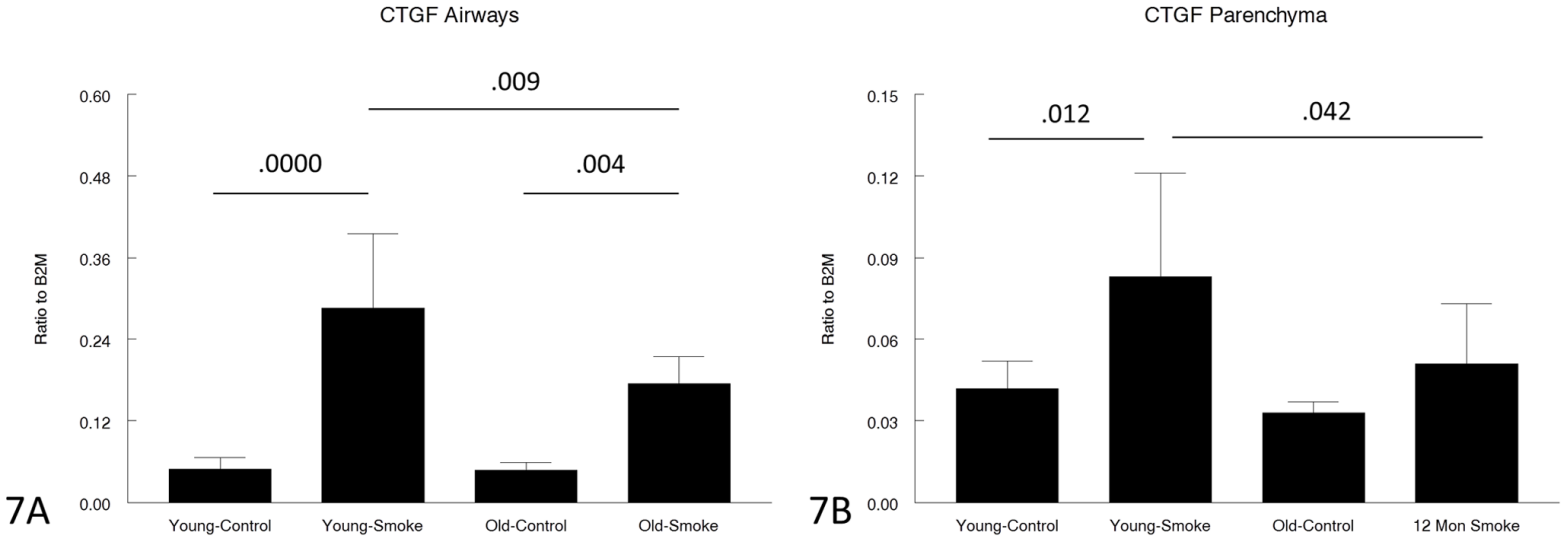
Gene expression of CTGF in laser capture microdissected airways (bronchioles) (7A) and parenchyma (7B). CTGF functions as a proximate mediator of TGFβ-induced matrix production, and is increased by smoke in both young and old animals, albeit to a lesser level in old animals. Data are mean ± SD. N = 5 animals/group. P values as shown.

**Figure 8:**
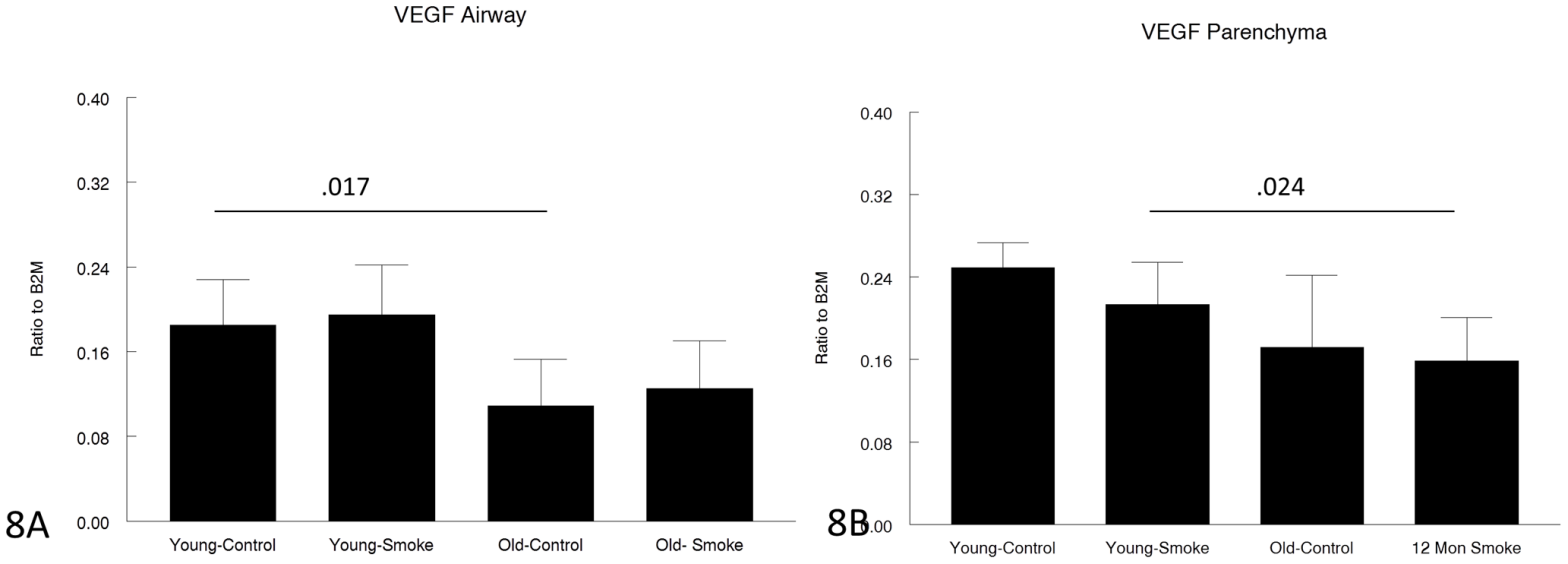
Gene expression of VEGF in laser capture microdissected airways (bronchioles) (8A) and parenchyma (8B). VEGF is believed to be a trophic factor for normal lung parenchymal maintenance and is decreased old compared to young animals. Data are mean ± SD. N = 5 animals/group. P values as shown.

**Figure 9:**
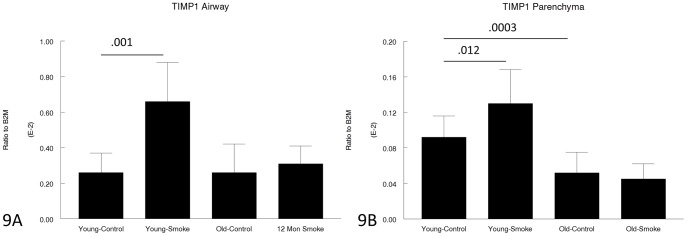
Gene expression of TIMP1 in laser capture microdissected airways (bronchioles) (9A) and parenchyma (9B). TIMP1 is upregulated by smoke in young animals, but not upregulated at all in old animals. Data are mean ± SD. N = 5 animals/group. P values as shown.

**Figure 10:**
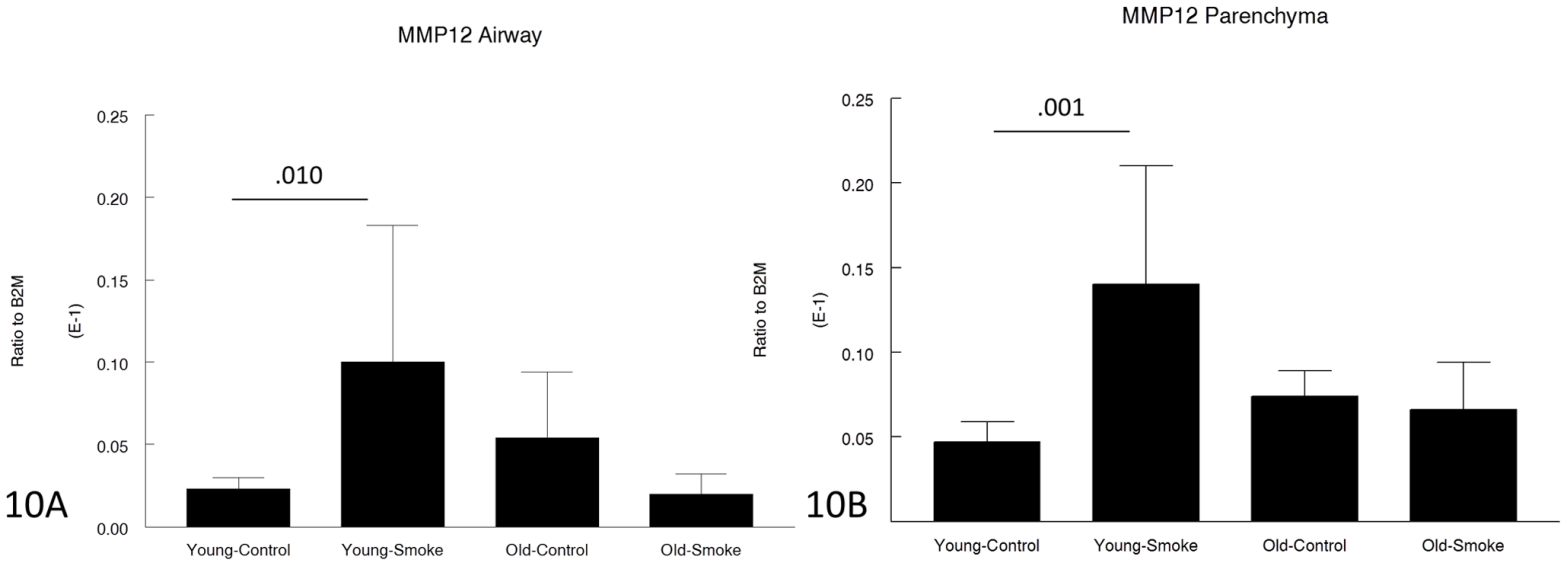
Gene expression of MMP-12 in laser capture microdissected airways (bronchioles) (10A) and parenchyma (10B). MMP-12, which appears play an important role in the pathogenesis of both emphysema and small airway remodeling (see text) is upregulated by smoke in young animals and not upregulated at all in old animals. Data are mean ± SD. N = 5 animals/group. P values as shown.

Expression of genes related to matrix production and/or breakdown is shown in Figures 5, 6, 7, 8, 9 and 10. In a very broad sense, expression levels of these genes tended to be lower in the 12 month animals, although for CTGF, which is a mediator of TGFβ1 driven airway remodelling [17] expression was increased in airways and parenchyma in both age groups, but to a somewhat lower level than in older animals (Figure 7). Interestingly, collagens 1 and 3 (Figures 5,6) tended to be decreased in airways in the 12 month group and showed a trend toward an increase in the airways in the 3 month group.

Some of the gene expression data were quite surprising; for example, MMP-12, which clearly plays an important role in both emphysema and small airway remodeling in the mouse since knockout of MMP-12 is protective against both lesions [18], [19], and which is increased by smoke exposure in 3 month animals, was not increased at all by smoke exposure in 12 month animals (Figure 10). On the other hand TIMP1, which would be expected to counter the effects of MMP-12, was increased in 3 month animals but not 12 month animals (Figure 9). Expression of VEGF, which may be crucial to maintenance of normal alveolar structure [20], was decreased somewhat in older animals (Figure 8).

Overall these data could be viewed as supporting a failure to repair the parenchyma in older animals, a process that has been postulated to lead to emphysema [19], but there is also a lack of over-expression of matrix in the small airways in older animals, so that they should, in theory, also be protected against remodeling, but in fact they are remodeled. Of interest, Huang et al [21], [22] reported that collagen as determined morphologically was increased in the small airways in normal old (20 month) compared to young (2 month) mice, and they also found that collagen 1α1 and 3α1 gene expression levels were decreased in the older animals (the comparison is somewhat tricky because they analysed whole lung and we analysed laser capture microdissected samples).

In practice, as noted above, no differences in airway remodeling or emphysema are seen between young and old, suggesting that in our study, as in those of Huang et al, these gene expression changes don = t translate readily to structural changes, and that in particular collagen actually deposited as matrix does not correlate well with collagen gene expression.

**Figure 11:**
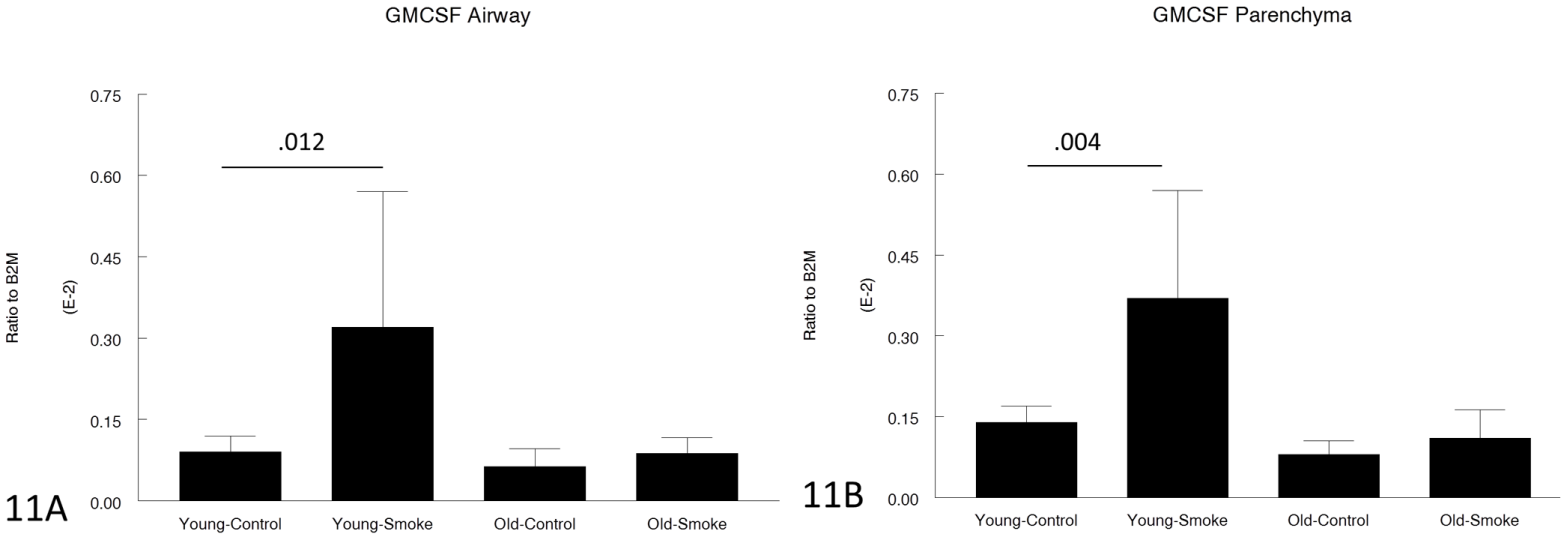
Gene expression of GM-CSF in laser capture microdissected airways (bronchioles) (11A) and parenchyma (11B). GM-CSF acts as an inflammatory cell chemoattractant in the lung and is upregulated by smoke in young animals, but not affected by smoke in old animals. Data are mean ± SD. N = 5 animals/group. P values as shown.

**Figure 12:**
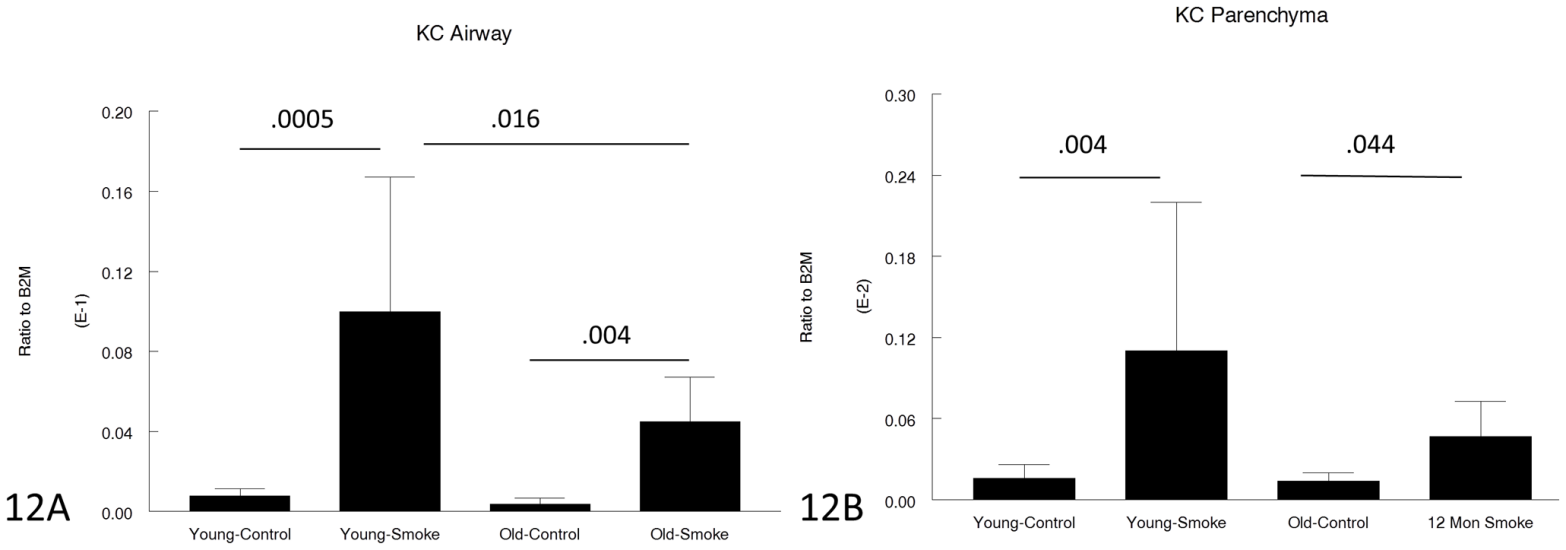
Gene expression of KC in laser capture microdissected airways (bronchioles) (12A) and parenchyma (12B). KC is a neutrophil chemoattractant and is upregulated by smoke in both young and old animals, but to a lesser extent in old animals. Data are mean ± SD. N = 5 animals/group. P values as shown.

**Figure 13:**
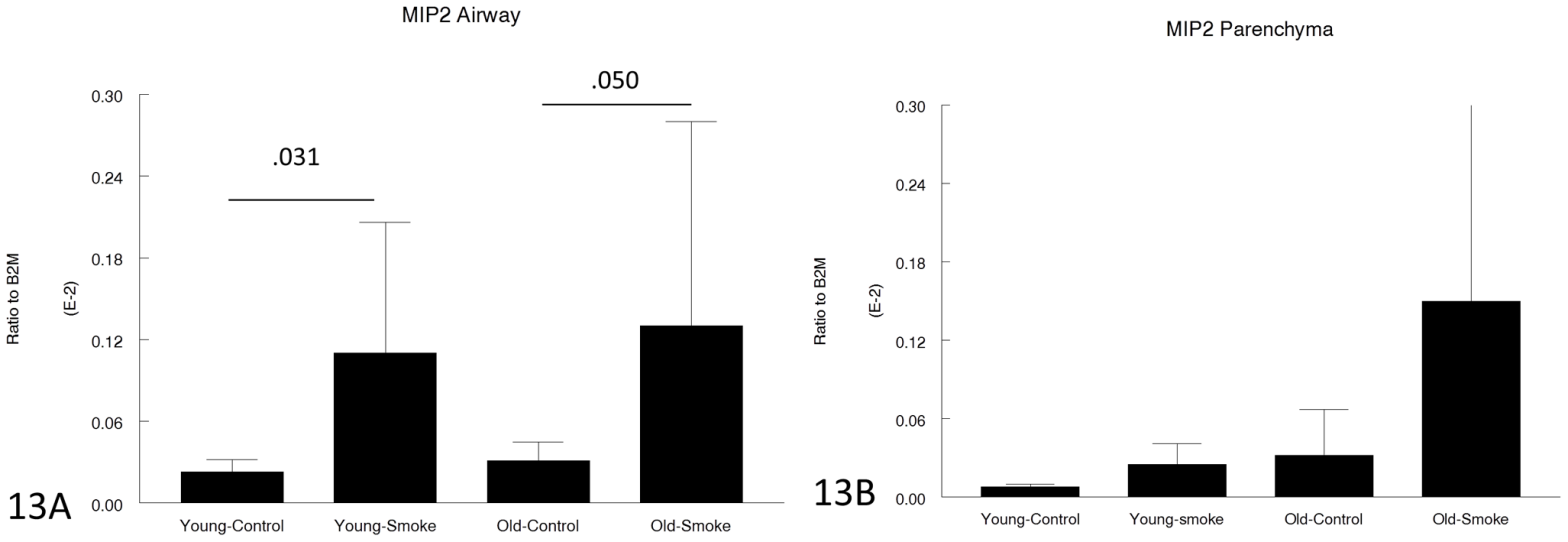
Gene expression of MIP-2 in laser capture microdissected airways (bronchioles) (13A) and parenchyma (13B). MIP-2 is a neutrophil chemoattractant and is upregulated by smoke in both young and old animals. Data are mean ± SD. N = 5 animals/group. P values as shown.

Figures 11–13 show expression of chemoattractant genes related to the inflammatory response. Neither KC nor MIP-2 was upregulated more in older compared to young animals (KC was in fact upregulated to a lesser degree in older animals) and GM-CSF was upregulated by smoke in young animals but not at all in older animals.

**Figure 14:**
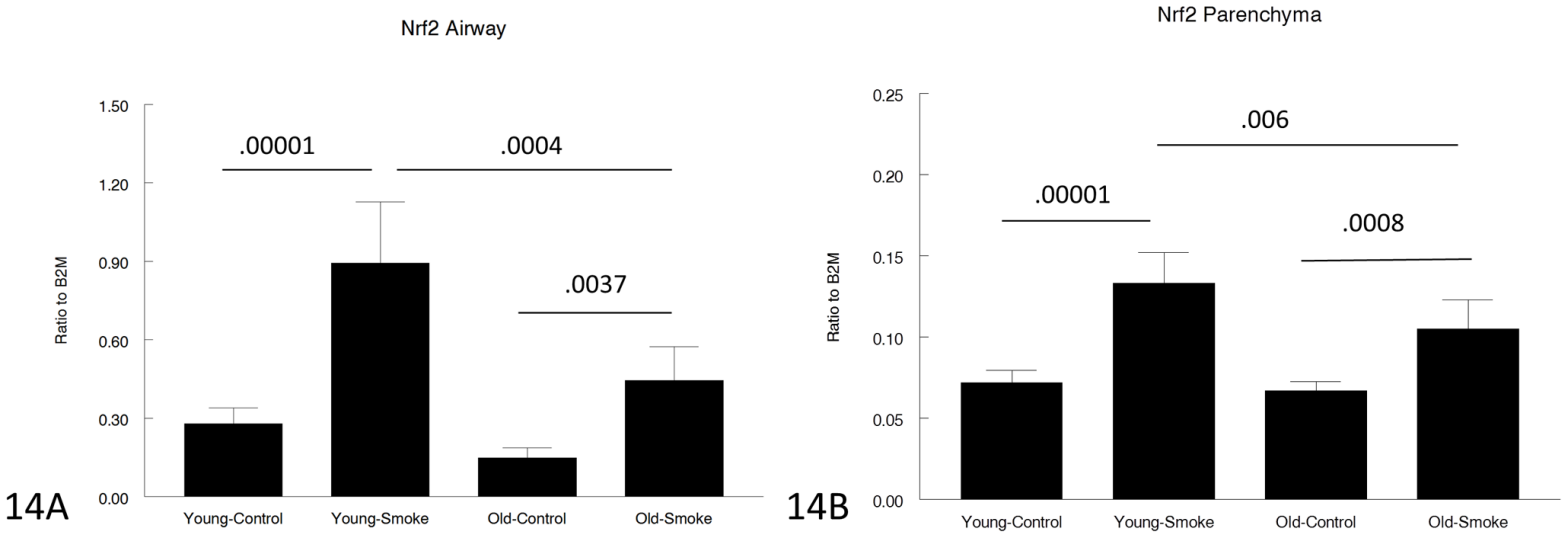
Gene expression of Nrf2 in laser capture microdissected airways (bronchioles) (14A) and parenchyma (14B). Nrf2 regulates the anti-oxidant response and is upregulated by smoke in both young and old animals, but to a lesser extent in old animals. Data are mean ± SD. N = 5 animals/group. P values as shown.

**Figure 15:**
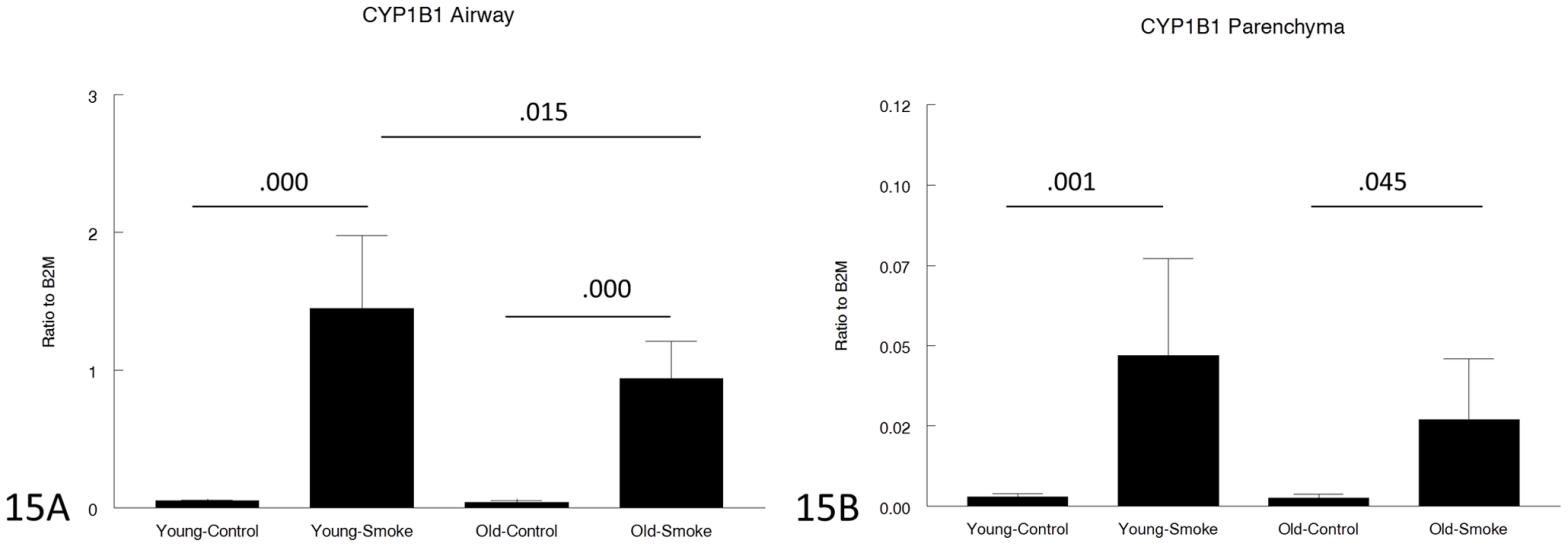
Gene expression of CYP1B1 in laser capture microdissected airways (bronchioles) (15A) and parenchyma (15B). CYP1B1 is a phase 1 detoxifying is upregulated by smoke in both young and old animals, but to a lesser extent in the airways in old animals. Data are mean ± SD. N = 5 animals/group. P values as shown.

**Figure 16:**
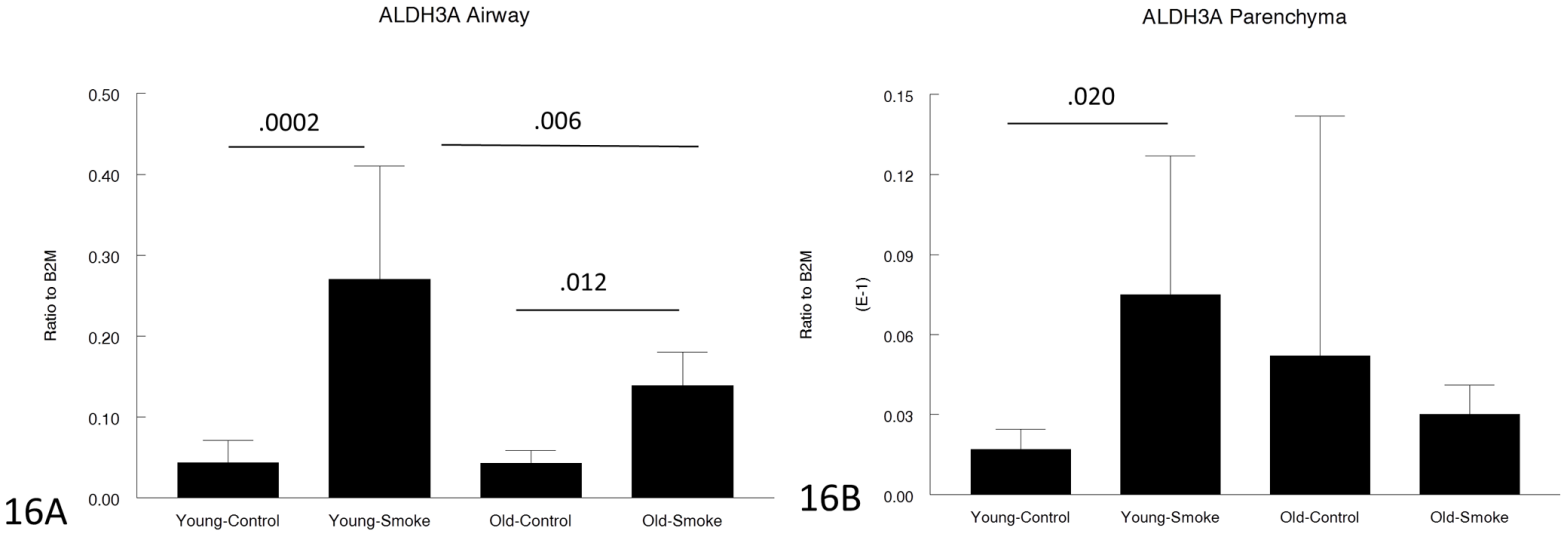
Gene expression of ALDH3A in laser capture microdissected airways (bronchioles) (16A) and parenchyma (16B). ALDH3A is a detoxifying enzyme and is upregulated by smoke in the airways in both young and old animals, but to a lesser extent in old animals, and not upregulated in the parenchyma in old animals. Data are mean ± SD. P values as shown.

**Figure 17:**
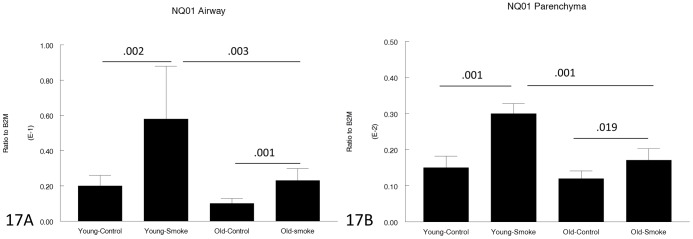
Gene expression of NQO1 in laser capture microdissected airways (bronchioles) (17A) and parenchyma (17B). **NQO1** is an anti-oxidant/free radical scavenger and is upregulated by smoke in both young and old animals, but to a lesser extent in old animals. Data are mean ± SD. N = 5 animals/group. P values as shown.

Figures 14–17 show genes involved in antioxidant and detoxification responses. The anti-oxidant regular, Nrf2 [23], was increased in airways and parenchyma in both 3 month and 12 month mice, albeit not to the same extent in the older animals. The same pattern was seen with the phase I enzyme, CYP1B1, and a variety of other antioxidant/detoxification genes (Figures 16, 17).

**Figure 18:**
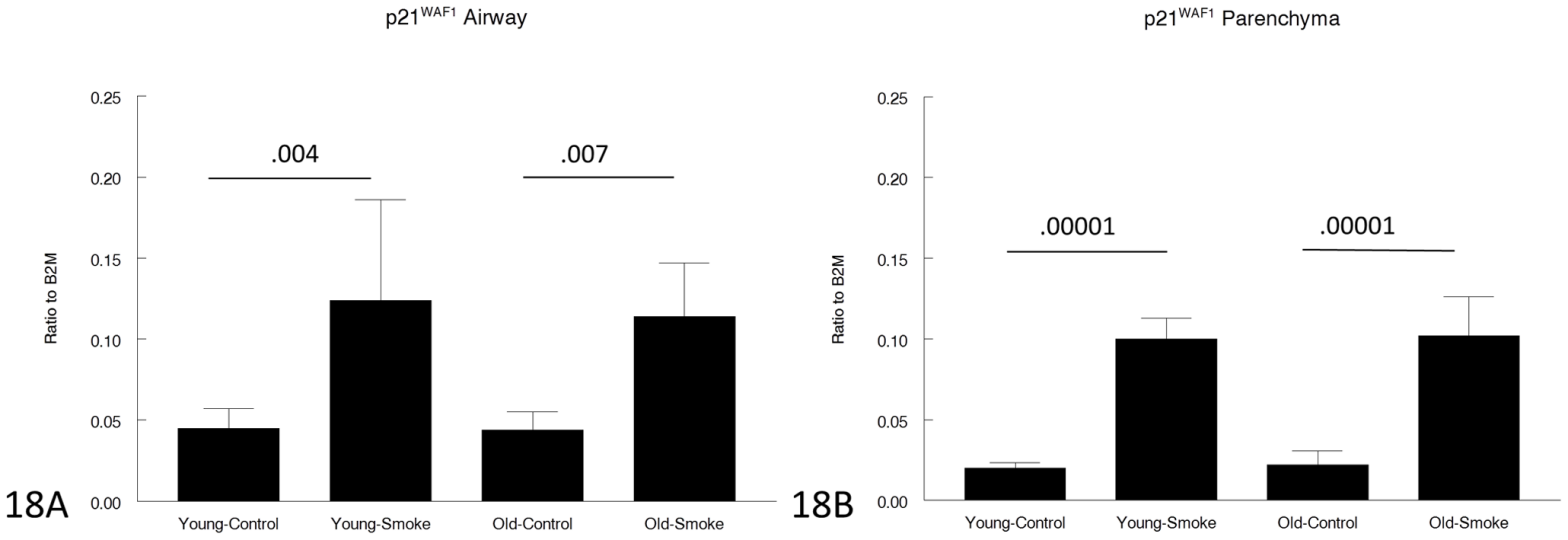
Gene expression of p21^WAF1^ in laser capture microdissected airways (bronchioles) (18A) and parenchyma (18B). p21^WAF1^ is believed to be a marker of senescence and is upregulated by smoke to the same extent in young and old animals. Data are mean ± SD. N = 5 animals/group. P values as shown.

**Figure 19:**
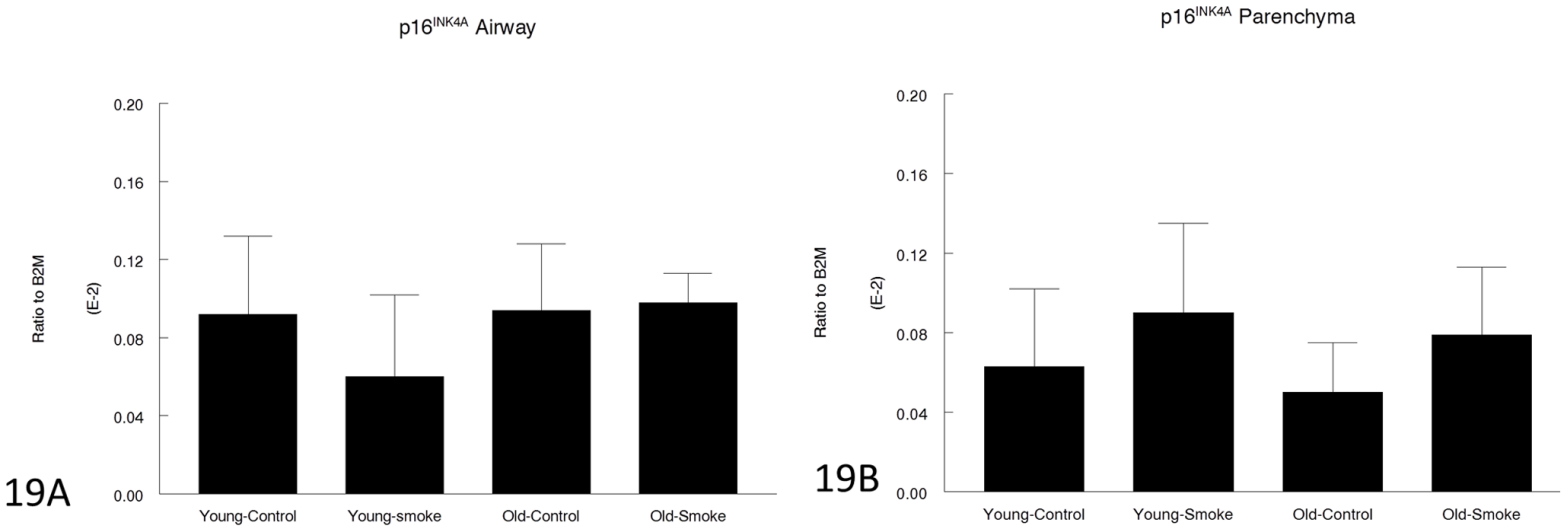
Gene expression of p16^INK4a^ in laser capture microdissected airways (bronchioles) (19A) and parenchyma (19B). p16^INK4a^ prevents cell cycle progression and is believed to be a marker of senescence. Expression is not statistically changes by smoke exposure in young or old animals. Data are mean ± SD. N = 5 animals/group. P values as shown.

Figures 18 and 19 show data for p21^WAF1^ and p16^INK4a^, genes believed to play a role in senescence. p16 gene expression was not significantly changed by age or smoke exposure. p21 expression in control animals was the same in young and old mice and was increased by smoke to the same extent at both ages.

### Smoking-induced and Age-related Telomere Shortening in Mice

**Figure 20:**
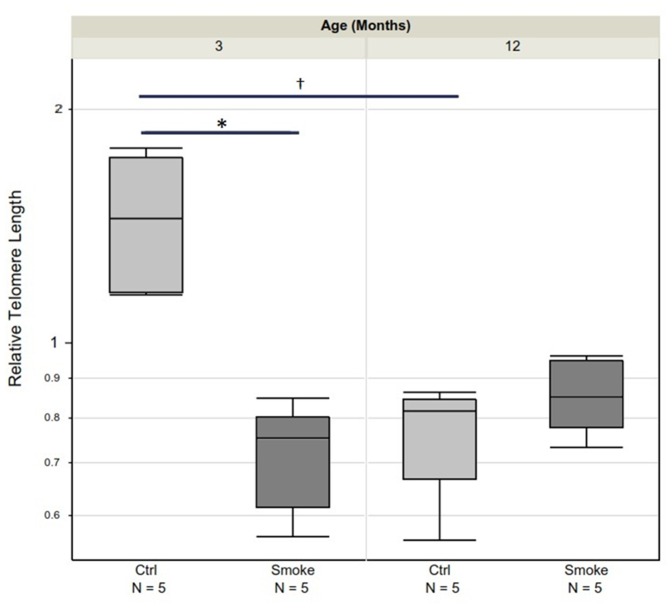
Comparison of telomere length between smoked and control mice segregated by age. Telomere length is greatest in young control animals and is decreased by smoke. Telomere length is also decreased by age but not affected by smoke in old animals. Data are shown as median and interquartile range (box). N = 5 animals/group. * P = 0.0055; † P = 0.0452.

Telomere length is a marker of aging/senescence. Relative telomere length (Figure 20), shown as median (IQR), for 3 month control, 3 month smoked, 12 month control, and 12 month smoked mice was 1.44 (1.16–1.73), 0.75 (0.61–0.80), 0.82 (0.67–0.85), and 0.85 (0.78–0.95), respectively. As expected, 12 month old control mice had shorter telomeres than the 3 month old control mice (P = 0.0452). Six months of smoke exposure significantly reduced telomere length in the 3 month but not 12 month old mice (p = 0.0055 in the 3 month old group).

## Discussion

A number of recent papers have advanced the idea that COPD represents a disease of accelerated lung aging/accelerated lung senescence. The evidence supporting this concept has been reviewed in detail elsewhere [1]–[6], but in brief, oxidative stress caused by cigarette smoke is believed to lead to cellular senescence, and this effect represents an exacerbation of normal aging, itself a process that has been suggested to result from slow free radical injury to tissue. Senescent cells lose the ability to replicate but instead generate a variety of inflammatory mediators; and ongoing inflammation, even in those who have stopped smoking, is characteristic of COPD [21]. Senescent cells can be identified by shortened telomeres, by immunohistochemical staining for senescence associated β-galactosidase, and by increased expression of p21^WAF1^ and p16^INK4a^ and all of these markers have been reported in the lungs of COPD patients compared to smokers without COPD or nonsmoking controls [1]–[6].

How aging/senescence leads to the anatomic changes of COPD is less clear, but one hypothesis is that, with time, replicating cells in the alveolar parenchyma are replaced by non-replicating inflammogenic senescent cells, so that normal lung maintenance of alveolar structure is impaired, and the destructive effects of proteases released from inflammatory cells cannot be repaired [2], [3]. In support of this idea, mice that lack telomerase develop enlarged airspaces (emphysema) [24], as do mice lacking senescence marker protein-30 [25], so-called senescence accelerated mice [26], or mice lacking the protein and histone deacetylase sirtuin 1 (SIRT1) [6], which is believed to protect against aging and oxidative stress. However, as pointed out by Fukuchi [26], absent cigarette smoke in these models there are enlarged airspaces but not an increased destructive index, suggesting these changes represent senile lung but not true emphysema.

Fukuchi [26] has suggested that the association of older age and COPD seen in humans could result from intrinsic development of COPD as a result of structural aging changes in the lung or from an enhanced susceptibility of the aged lung to external insults such as cigarette smoke. One test of this general hypothesis is to look at the effects of cigarette smoking in young versus old laboratory animals, but there is surprisingly little in the literature on this question, or in fact on the question of whether older animals generally mount different inflammatory and antioxidant responses compared to younger animal.

Using mice exposed only to air and not smoke, Aoshiba and Nagai [9] found 8 genes that were upregulated in the lungs of 24 month old compared to 12 week old Balb/C mice; these genes were proinflammatory and primarily related to T lymphocyte chemoattraction. In the same study, there was a considerable increase in CD4 and CD8 T lymphocytes and macrophages in the lungs of the older animals. Calvi et al [10] reported a somewhat similar result, with, in addition, increased evidence of oxidative stress and metalloprotease activity in DBA/2 mice that developed between 8 and 12 months of age (although, oddly, these effects regressed by 20 months). Aoshiba and Nagai [3] have suggested that senescence of Clara cells leads to airway remodeling and experimentally this effect can be produced by repeated administration of the Clara cell toxicant naphthalense [7], although there does not appear to be a direct test of this idea in old vs young animals.

Moriyama et al [8] compared 9 week old and 69 week old C57Bl/6 mice that were exposed to cigarette smoke once or for 9 consecutive days and found that the older animals had a greater neutrophil influx, higher levels of the neutrophil chemoattractants MIP-2 and KC, and greater nuclear translocation of NF-κB. There were no age related differences in gene expression levels of antioxidant and detoxifying enzymes. Gould et al [11] looked at the effect of age on the smoke-induced glutathione response in the lung using C57Bl/6 mice age 2, 8, 13, 19, and 26 months. Smoke-induced increases in lung lining fluid GSH were greatest in 2 month old animals and considerably less in all the older animals, implying that older animals are not able to mount as effective an antioxidant defence as younger animals.

However, not all studies of young and old animals have shown a similar pattern. Several investigators [27]–[30] have examined the effects of ozone, another powerful oxidant, in mice and rats and found that older animals showed either lesser functional decrements and lesser inflammatory responses compared to younger animals or no differences. Shore et al [27] found that levels of soluble TNF receptor 1 were increased in the older animals, and suggested that that this phenomenon might account for the decreased inflammatory response.

In the present study we have looked at this question somewhat differently, asking whether there are differences in inflammatory response and oxidant damage as well as in expression of a variety of genes believed to play a role in COPD in young versus old mice, and, if so, whether these differences translate into differences in disease expression. The answer to the first question is that there are only minimal differences in inflammatory response, at least in terms of neutrophils and macrophages in tissue, with a slightly lesser neutrophil response in older animals. Overall there were no consistent differences in expression of pro-inflammatory mediators. In fact, for GM-CSF, which functions as an inflammatory chemoattractant in the lung [31] older animals showed no response to cigarette smoke at all, and for the neutrophil chemoattractant, KC, responses to smoke were lower in the old compared to the young animals, whereas for another neutrophil chemoattractant, MIP-2, responses were largely the same.

For genes involved in the antioxidant/detoxification response such as the master antioxidant response regulator, Nrf2 [23], [32] and the phase I detoxifying enzyme, CYP1B1, there was a trend toward a lesser increase after smoke exposure in the old compared to the young animals (Figures 14–17), but for all antioxidant/detoxification genes for which smoke caused an upregulation of expression in young animals, there was also an upregulation of response in old animals. Most important, using 8-hydroxyguanosine as a marker of oxidant damage, the proportion of 8-hydroxyguanosine positive airway epithelial cells was exactly the same in young and old animals, suggesting that whatever differences there are between young and old, they are not sufficient to increase oxidant damage in old animals.

Our results thus differ from those reported by Moriyama et al [8] even though both laboratories were using old C57Bl/6 mice that were about the same age at the end of the experiment (69 weeks for Moriyama et al; 78 weeks here). One potential reason for this difference is that Moriyama et al were using single or short term smoke exposures whereas we were using very long term exposures, and it is possible that with chronic exposures the response to smoke is modified. Along this line, Ragasawamy [33] demonstrated that with a single exposure to cigarette smoke, many genes are upregulated in mouse lungs, but when the smoke exposures last for 6 months, the numbers of upregulated genes is far smaller.

The response of mice to cigarette smoke also varies considerably from strain to strain [34] and another possibility is that even though both our laboratory and Moriyama et al [8] were notionally using C57Bl/6 mice, mice sold as C57Bl/6 from different vendors may not really be genetically the same, as we have suggested elsewhere [13].

Our results also suggest that the concept of senescence driving COPD, or at least emphysema, is not simple. It is true that p21^WAF1^, which is often regarded as a senescence marker [1]–[6] is upregulated in our animals, but it is upregulated to exactly the same degree in young and old animals and in airways and parenchyma. Conversely, expression of p16^INK4a^, which is thought to maintain cells in a nonreplicating senescent state [1], and which some reports indicate is upregulated in smoke-exposed mice [6], was not upregulated at all in our study. Telomere length was significantly reduced by cigarette smoke in the young but not in the older mice. This raises the possibility that while accelerated replicative senescence (as reflected by telomere measurements) may be important in the pathogenesis of COPD in young mice, it may play a lesser (and perhaps no significant) role in older mice. As suggested by Tuder [2], senescence probably needs to be separated from age.

The most important conclusion from our study is that there are no differences in terms of morphologic outcome in young vs old animals: both develop the same degree of emphysema and both develop the same degree of small airway remodeling, and since we have shown elsewhere that these measures correlate well with pulmonary function in laboratory animals [35], it is fair to conclude that, in this set of experiments, age does not influence the severity of smoke-induced COPD. These results suggest that, in contrast to the scenario seen with markedly abrogating the anti-oxidant response, for example by completely deleting Nrf2 [23], the somewhat lower antioxidant response found here in older mice does not translate directly into greater severity of disease. The limits of these conclusions need to be stressed, however, because it is possible that if the experiments were allowed to run for the full length of a C57Bl/6 lifetime of about 2 years, a lowered antioxidant response might produce differences in disease severity.
